# Difficult colonoscopy score identifies the difficult patients undergoing unsedated colonoscopy

**DOI:** 10.1186/s12876-015-0273-7

**Published:** 2015-04-09

**Authors:** Hui Jia, Limei Wang, Hui Luo, Shaowei Yao, Xiangping Wang, Linhui Zhang, Rui Huang, Zhiguo Liu, Xiaoyu Kang, Yanglin Pan, Xuegang Guo

**Affiliations:** 1 Xijing Hospital of Digestive Diseases, Fourth Military Medical University, Xian, China; 2 Shannxi Second People’s Hospital, Xian, China

## Abstract

**Background:**

Many factors have been found to affect the difficulty of colonoscope insertion, such as age, gender, body mass index (BMI), history of abdominal surgery and operator etc. However, a scoring system may be more useful to predict the difficulty during colonoscopy.

**Methods:**

The individual and procedure-related data of 616 patients undergoing colonoscopy were prospectively collected from December 2013 through February 2014 in Xijing Hospital of Digestive Diseases. Cox regression analysis was used to identify high-risk factors associated with difficulty of colonoscopy. A predicting model with the difficult colonoscopy score (DCS) was developed.

**Results:**

Total cecum intubation rate was 98.9% (609/616). Advanced age, lower BMI, inexperienced operator and fair or poor sleep quality were identified as independent factors of prolonged insertion time (all p < 0.05), which were used to develop the DCS. Based on the score, patients could be divided into high-risk and low-risk groups with distinct incomplete rates within 10 min (42.0% vs. 16.5%, p < 0.001). Compared with those with DCS ≤ 1, patients with DCS > 1 had increased insertion time (10.6 ± 0.7 min vs. 6.9 ± 0.2 min, p < 0.001) and pain score (1.9 ± 1.5 vs. 1.4 ± 1.4, p = 0.002). More abdominal compression (36.9% vs. 16.8%, p < 0.001) and position change (51.4% vs. 22.6%, p < 0.001) were needed in this group of patients.

**Conclusion:**

Patients with DCS > 1 had longer insertion time, higher pain score and needed more abdominal compression and position changes. DCS was useful for predicting the difficulty of colonoscope intubation.

(ClinicalTrials.gov NCT02105025 05/05/2014).

**Electronic supplementary material:**

The online version of this article (doi:10.1186/s12876-015-0273-7) contains supplementary material, which is available to authorized users.

## Background

Colonoscopy is widely used for management of colorectal diseases. Several indicators reflect the performance quality of colonoscopy, including adenoma detection rate, adverse events rate, withdrawal time and cecal intubation rate [1,2]. A high rate of cecal intubation is necessary for achieving a complete and thorough examination of the colon. According to the recommendations of the US Multi-society Task Force on Colorectal Cancer, cecal intubation rate above 90% in all examinations and above 95% in screening colonoscopy should be achieved by endoscopists [3].

Although completion rates have been reported as more than 95% in many studies [4,5], colonoscopists do meet difficulties during colon insertion in some situations. It often needs tremendous efforts and prolonged insertion time in difficult patients. Although there is no standard definition of difficult colonoscopy, procedures with more than 10 min for insertion or at least two attempts to reach the cecum, or finally failed intubation are often considered difficult [6,7]. Because nearly all of the procedures of failed intubation or several attempts for insertion take at least 10 min, prolonged insertion time (>10 min) seems to be an appropriate and quantitative surrogate of insertion difficulty.

Several studies has revealed that some variables are risk factors of difficulty of colonoscopy, including gender, age, obesity, bowel preparation, and history of abdominal and/or pelvic surgery and complicated diverticular disease etc [7-13]. Difficulty of colonoscopy may be determined by a combination of these factors. Nakamura et al. proposed a scoring system base on these factors could be calculated prior to the procedure in order to prediction of difficult colonoscopy [14]. However, it was a pilot study and only 30 patients were enrolled.

Here we prospectively collected the data of insertion during colonoscopy and investigated the possible risk factors associated with prolonged insertion time by multivariate regression analysis. Furthermore, we developed a scoring system to predict the difficulty of colonoscopy.

## Methods

### Patients

This prospective study was conducted in the Endoscopy Center of Xijing Hospital of Digestive Diseases in China. Consecutive patients aged 18–90 years old who underwent unsedated colonoscopy were enrolled. Exclusion criteria included: (1) no bowel preparation or colon cleansing by enema only; (2) unnecessary to reach cecum; (3) prior finding of severe colon stenosis or obstructing tumor; (4) history of colectomy; (5) unstable hemodynamics; (6) pregenancy; (7) unable to give informed consent.

Written informed consent was obtained from all the patients. The study protocol and informed consent form were approved by the institutional review board of Xijing Hospital. This study was registered with Clinical Trials.gov (NCT02105025 05/05/2014).

### Bowel preparation and unsedated colonoscopy

All patients were prescribed polyethylene glycol electrolyte powder (PEG-ELP, each sachet containing 59 g polyethylene glycol 4000, 1.46 g sodium chloride, 5.68 g sodium sulfate, 0.74 g potassium chloride, 1.68 g sodium bicarbonate; WanHe Pharmaceutical Co, Shenzhen, China) or sodium phosphate (Fleet Phospho-soda; CB Fleet Company, Switzerland) for bowel preparation according to the preference of physicians. They were asked to drink two bags of PEG-ELP dissolved in 2 L of water, or 45 mL of sodium phosphate be diluted in 240 mL of cool water following with at least 1.5 L of water at 05:00–06:00 h within 2 h on the day of colonoscopy. Patients were encouraged to drink more clear liquids after purgatives for adequate hydration before colonoscopy. In addition, patients were instructed to have a regular meal for lunch and only liquid diets for dinner the day before the operation. This preparation method had previously reported with acceptable cleansing rate, tolerance and polyp detection rate [15-18]. The quality of bowel preparation was evaluated by Ottawa scoreduring withdrawal of colonoscopy as described previously [19].

All colonoscopies were performed at 08:00–13:00 AM, 18 colonoscopists participated in this study and were categorized as senior if they had performed 1000 or more colonoscopies independently and junior if they had performed less than 1000 colonoscopies independently. The Fujinon colonoscope (CV-240, Japan) was used for every procedure. Air was insufflated during insertion and withdrawal.

### Data collection and outcomes measurement

Demographic data and clinical characteristics of all patients were collected. The degree of maximum abdominal pain during the procedure was assessed by using visual analog scale (VAS) with 10-point scale (1 = no pain and 10 = severe and intolerable pain). Anxiety was evaluated by Hospital Anxiety and Depression scale (HAD) as described previously [20]. Sleep quality was collected through questionnaires by interviewing patients before the procedure by a special staff, which was categorized as excellent or good, fair or bad as described previously [15,21]. During scope intubation, the maneuvers of abdominal compression and position changes were recorded. Cecal insertion time was recorded from the beginning of insertion to visualization of any of the following anatomical landmarks: ileocecal valve, appendiceal orifice or terminal ileum. If any doubt existed, the colonoscopy was defined as incomplete. All data were collected by one investigator (WLM) who did not participate in data analysis.

### Statistical analysis

As an event-driven longitudinal procedure, Kaplan-Meier analysis provides a better means of assessment for determining the period than cross-sectional tests [22]. In the present study, only patients with the scope insertion to cecum were defined as “censored cases” (=success). Others were defined as termination due to “failure” (=failure). Log-rank test was used to assess the effect of single variable on insertion time. To adjust confounding factors, multivariate analysis (Cox regression analysis) was used for those covariates with p values of <0.1 in single factor analysis. A DCS was developed in line with the regression coefficients of the significant variables of multivariate regression analysis. The cutoff values of DCS were determined by receiver operator characteristics (ROC) analysis of cecal intubation completed within 10 min. Continuous variables were expressed as means with standard deviation (SD) and analyzed with Student’s t test or one-way ANOVA. Categorical variables were analyzed using chi-square test or Fisher exact test when appropriate. Analyses were performed with SPSS V.17.0 for Windows (IBM). All reported p-values were results of two-side test and those <0.05 was considered to be significant.

## Results

### Baseline of patient characteristics

**Figure 1:**
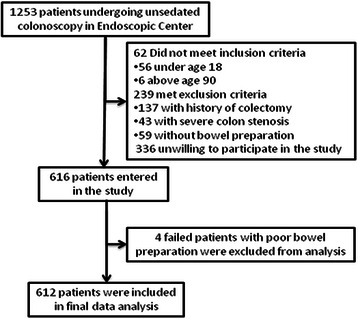
**Flowchart of the study.**

From December 2013 to February 2014, a total of 1253 patients undergoing unsedated colonoscopy were prospectively enrolled in our endoscopy center. 637 patients were excluded, among which 62 did not meet inclusion criteria (including 56 less than 18 years old and 6 above 90), 239 met exclusion criteria (including 137 with history of colectomy, 59 without bowel preparation and 43 with severe colon stenosis) and 336 denied to participate in this study. Finally 616 patients entered in this study. Total cecum intubation rate was 98.9% (609/616), with 7 patients failed because of poor bowel preparation (n = 4) and technical difficulty (n = 3). The 4 failed patients with poor bowel preparation were excluded from final data analysis (Figure 1). The mean age of the study population was 50.4 ± 14.0 years, 315 (51.5%) were males and 500 (81.7%) received education of high school or above (Table 1). For the patients with successful intubation, the mean insertion time was 7.4 ± 5.2 min. 20.9% of patients had an insertion time of more than 10 min. There was no complications were found in all patients.

**Table 1:** **Baseline of patient characteristics**

Clinical features	Patients (n = 612)
Age (years)	50.4 ± 14.0
Gender	
Male	315 (51.5%)
Female	297 (48.5%)
BMI(kg/m2)	22.7 ± 3.7
Grade of education	
Elementary or below	112 (18.3%)
High school or above	500 (81.7%)
Marriage status	
Single	34 (5.6%)
Married	578 (94.4%)
Smoking	126 (20.6%)
Drinking	136 (22.2%)
Patients type	
Outpatient	486 (79.4%)
Inpatient	118 (19.3%)
Emergency	8 (1.3%)
Previous surgery(abdominal and/or pelvic)	162 (26.5%)
Diverticulosis	10 (1.6%)
HAD	2.6 ± 4.4
VAS	1.5 ± 1.4
Symptoms	
Constipation	99 (16.2%)
Abdominal pain	175 (28.6%)
Diarrhea	98 (16.0%)
Others	172 (28.2%)
Sleep quality	
Excellent or good	534 (87.3%)
Fair or bad	78 (12.7%)
Indication for colonoscopy	
Screening or surveillance	137(22.4%)
Diagnosis	475(77.6%)
Interval time from appointment to colonoscopy (days)	8.5 ± 6.9
Purgative type	
PEG-ELP	550(89.9%)
SP	62(10.1%)

BMI, body mass index; HAD, Hospital Anxiety and Depression Scale; VAS, Visual Analogue Scale PEG-ELP, polyethylene glycol electrolyte powder; SP, sodium phosphate.

### Factors associated with insertion time by univariate and multivariate analysis

By univariate analysis (Table 2), the mean insertion time was found to be shorter in patients less than 65 years old than those more than 65 (7.4 ± 0.3 min vs. 9.0 ± 0.6 min, p = 0.020). Men had a shorter insertion time than woman (7.1 ± 0.3 min vs. 8.2 ± 0.4 min, p = 0.019). Colonoscopies (n = 198) performed by junior endoscopists (n = 9) required longer insertion time than colonoscopies (n = 414) performed by seniors (n = 9) (p = 0,004). BMI were significantly associated with the insertion time to cecum (p < 0.001). According to the Kaplan-Meier curves (Additional file 1: Figure S1), the 3 higher BMI were combined for further multivariate regression analysis. Patients with fair or bad sleep quality had a longer insertion time than the others (p = 0.004). Smokers tended to have shorter insertion time than nonsmokers although the difference was not significant (p = 0.089). If the interval time of appointment-to-colonoscopy was less than 10 days, the insertion time tended to be shorter (p = 0.065). There were no differences of insertion time regarding patients with different education levels, marital status, symptoms, history of surgery, anxiety (by HAD score), patient types and indications of colonoscopy etc.

**Table 2:** **Univariate analysis of factors associated with insertion time duringunsedated colonoscopy**

Variable	N	Insertion time (min)	p value
Age (years)			
<65	518	7.4 ± 0.3	
≥65	94	9.0 ± 0.6	0.020
Gender (male/female)			
Male	315	7.1 ± 0.3	
Female	297	8.2 ± 0.4	0.019
BMI (kg/m2)			
Underweight (<18.5)	68	10.8 ± 1.0	
Healthy weight (18.5-24.9)	383	7.3 ± 0.3	
Overweight (25.0-29.9)	145	7.2 ± 0.5	
Obesity (≥30)	16	6.4 ± 0.9	<0.001
Colonoscopists			
Senior(n = 9)	414	7.2 ± 0.3	
Junior(n = 9)	198	8.5 ± 0.4	0.004
Sleep quality			
Excellent or good	534	7.3 ± 0.2	
Fair or bad	78	9.7 ± 1.0	0.004
Interval time of appointment to colonoscopy (days)			
<10	348	8.0 ± 0.3	
≥10	264	7.1 ± 0.3	0.065
Smoking			
Yes	126	7.0 ± 0.5	
No	486	7.7 ± 0.3	0.089

BMI, body mass index.

Table 3 provided an outline of multivariate analysis. Of all the factors associated with insertion time (p < 0.1) found by univariate analysis, only four factors had independent impact on insertion time during colonoscopy. Colonoscopy performed by junior colonoscopists (HR 1.29; 95% CI, 1.08-1.54; p = 0.004), patients with fair or bad sleep quality (HR 1.33; 95% CI, 1.04-1.72; p = 0.026), those with BMI < 18.5 (HR 1.59; 95% CI, 1.23-2.07; p = 0.001) or age ≥ 65 (HR 1.29; 95% CI, 1.03-1.62; p = 0.027) were independently associated with longer insertion time.

**Table 3:** **Multivariate analysis of factors associated with insertion time during unsedated colonoscopy**

Variables	N = 612	HR (95% CI)	p value	B score	DCS points
Age (years)					
<65	518				
≥65	94	1.29 (1.03-1.62)	0.027	0.26	1
BMI (kg/m2)					
≥18.5	544				
<18.5	68	1.59 (1.23-2.07)	0.001	0.47	2
Colonoscopists					
Senior	414				
Junior	198	1.29 (1.08-1.54)	0.004	0.26	1
Sleep quality					
Excellent or good	534				
Fair or bad	78	1.33 (1.04-1.72)	0.026	0.29	1

BMI, body mass index.

### Deviation of a predicting model to predict difficult colonoscopy

To facilitate establishing a prediction model of insertion time of colonoscopy, the regression coefficients (B-score) of four independent factors were multiplied by 4 and rounded. Thus, DCS = 1 × A (1 if age ≥ 65y, 0 if <65y) + 2 × B (1 if BMI < 18.5, 0 if ≥18.5) + 1 × C (1 if colonoscopist is junior, 0 if senior) + 1 × S (1 if sleep quality was fair or bad, 0 if excellent or good) (Table 3). Then, we got a 6 point scoring system (0–5 point), and we found as the score rose the insertion time prolonged and the difficult rate increased. From 0–5 point, the insertion time was 6.4 ± 5.1, 7.5 ± 5.0, 9.9 ± 7.0, 11.7 ± 9.2, 14.0 ± 5.3, 15.0 ± 11.0 respectively, p = 0.006. The rate of insertion time of more than 10minwas 12.4%, 21.1%, 35.4%, 47.4%, 60.0%, 66.7% respectively, p < 0.001 (Table 4.). Next, we performed a ROC curve, the area under the ROC for predicting difficult colonoscopy (insertion time more than 10 min) was 0.66, with an optimal threshold of 1 point (Additional file 2: Figure S2). The sensitivity, specificity, positive and negative predictive value of DCS > 1 for the prediction of difficult colonoscopy was 73%, 50%, 85% and 66% respectively. Base on ROC curve, patients could be divided into low-risk(DCS ≤ 1) and high-risk (DCS > 1). Compared with low-risk patients, the difficult rate in high-risk patients with DCS > 1 was higher (16.5% vs. 42.0%, p < 0.001).

**Table 4:** **The effects of different DCS on the colonoscopy**

	DCS value						
	0	1	2	3	4	5	P value
Insertion time (min)	6.4 ± 5.1	7.5 ± 5.0	9.9 ± 7.0	11.7 ± 9.2	14.0 ± 5.3	15.0 ± 11.0	0.006
Difficult rate	12.4%	21.1%	35.4%	47.4%	60.0%	66.7%	<0.001

DCS, difficult colonoscopy score.

### Effect of different DCS on the difficulty-related variables of colonoscopy

We further analyzed the difference of insertion time, maximal pain sore and the need of abdominal compression and position changes in patients with different DCS (Table 5). Patients with DCS > 1 had a significantly longer insertion time compared with those with DCS ≤ 1 (10.6 ± 0.7 min vs. 6.9 ± 0.2 min, p < 0.001). The maximal pain score in patients with DCS > 1 was 1.9 ± 1.5 which was higher than 1.4 ± 1.4 in those with DCS ≤ 1 (p = 0.002). More abdominal compression (36.9% vs. 16.8%, p < 0.001) and position changes (51.4% vs. 22.6%, p < 0.001) were found in patients with DCS > 1.

**Table 5:** **The effects of different DCS groups on the procedure of colonoscopy**

	DCS > 1 (n = 111)	DCS ≤ 1 (n = 501)	p value
Mean insertion time (min)	10.6 ± 0.7	6.9 ± 0.2	<0.001
Maximal pain score	1.9 ± 1.5	1.4 ± 1.4	0.002
Abdominal compression (%)	41 (36.9%)	84(16.8%)	<0.001
Position change (%)	57(51.4%)	113(22.6%)	<0.001

DCS, difficult colonoscopy score.

### DCS for prediction of difficulty in subgroups of patients

**Figure 2:**
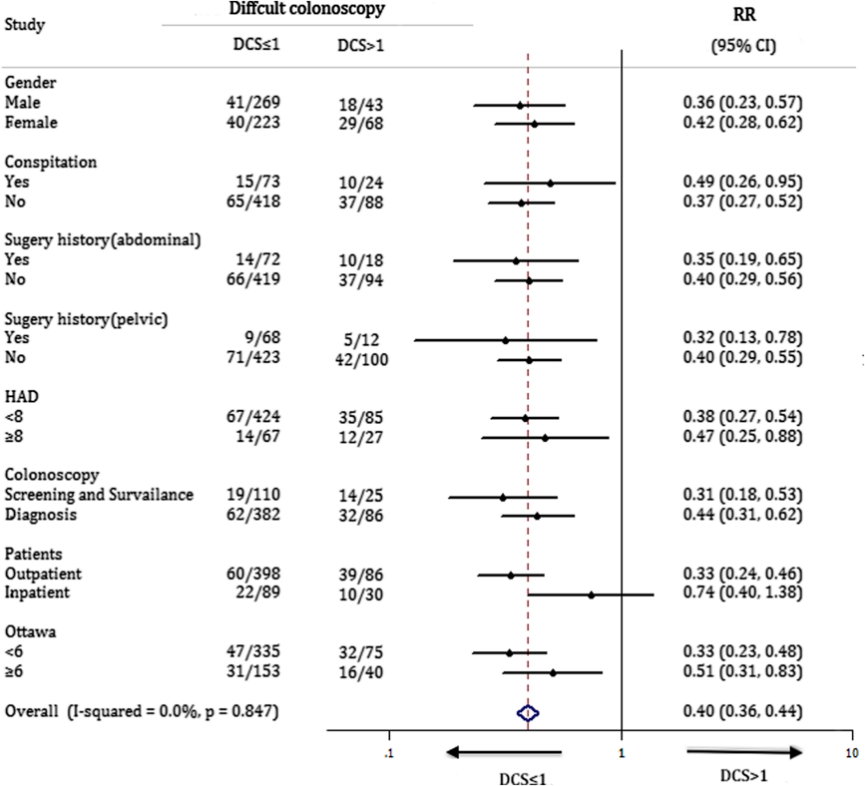
**Subgroup analysis for difficult colonoscopy in subjects of this study.** Difficult colonoscopy was defined by the procedure with insertion time more than 10 min. The effects of distinctDCS (≤1 vs. >1) on cecal intubation completed more than 10 min were analyzed in subgroups of patients, which were stratified by gender, presence of constipation, prior history of abdominal or pelvic surgery, anxious status, patient types, indications of colonoscopy and quality of bowel preparation.

The effects of different DCS (≤1 vs. >1) on difficulty of colonoscopy in subgroups of patients were further evaluated. The stratified factors included gender, presence of constipation, prior history of abdominal or pelvic surgery, anxious status (by HAD score), patient types, indications of colonoscopy and quality of bowel preparation. As shown in Figure 2, colonoscopy tended to be more difficult with longer insertion time in patients with DCS > 1. This result was consistent nearly for all subgroups of patients (except for inpatients).

## Discussion

It has been estimated that complete intubation of the colon is considerably difficult in up to 10-20% of procedures [23]. The difficulty of insertion during colonoscopy is largely related to looping of the colonoscope which displaces the colon from its native configuration. It is important to identify the potentially difficult cases before colonoscopy. Special intubation techniques or strategies, such as water-aided or cap-assisted method [24,25], single or double balloon enteroscopy [26-29] or magnet-imaging enhanced colonoscopy [8] etc. can be used early in these groups of patient to avoid of excessive insertion time, increased discomfort and even unnecessary adverse events. In the present study, by using insertion time as a surrogate and quantitative endpoint of difficulty, four independent variables, including age, BMI, case volume of colonoscopists and sleep quality were identified as high-risk factors associated with intubation difficulty. Based on these factors, we developed a DCS to predict the prolonged insertion time and difficulty of colonoscopy. High-risk patients with DCS > 1 had an increased mean insertion time (1.5 times) and pain score (1.4 times) and needed more abdominal compression (2.2 times) and position changes (2.3 times). For the patients with DCS > 1, several strategies can be chosen, including appointment with senior endoscopist or intubating with pediatric scope [30]. Some techniques may also be used to facilitate scope insertion, such as water-aided or cap-fitted or magnet-imaging enhanced method.

Several studies had revealed that some factors were related to the difficulty of colonoscope intubation, including advanced age, lower BMI, technical skill of the endoscopist, female gender, presence of constipation, history of abdominal or pelvic surgery, and inadequate bowel preparation [10,11,31,32]. However, the results among these studies were inconsistent. In the present study, we confirmed that the former three factors were associated with the insertion difficulty whereas the others were not. The different findings of risk factors among the studies may be mainly due to different study design, enrolled population, definitions of difficulty and the indications of colonoscopy. Regardless of the possibly other risk factors, the effectiveness of DCS was found to be consistent across nearly all subgroups of patients (Figure 2).

The present study revealed that sleep quality was an independent predictor of prolonged insertion time, which had not been examined in previous studies. Patients with fair or bad sleep quality was shown to have a longer insertion time. However, the reasons why sleep quality can affect the difficulty of colonoscopy are not clear. Some evidences suggested that the feeling of pain was related to sleep quality, with poor quality of sleep often independently associated with greater pain sensitivity [33]. In this study, the maximal pain score rated by patients with fair or bad sleep quality tended to be higher than those with excellent or good (1.7 ± 1.4 vs. 1.5 ± 1.4, p = 0.33), although the difference was not significant. The difficulty of colonoscope intubation may be increased due to the poor tolerance of pain or discomfort in patients with poor sleep quality, especially in the situation of unsedated colonoscopy. Furthermore, it had been found that patients with fair or bad sleep quality tended to have inadequate bowel preparation in our previous study [15], which might also increase the difficulty of scope insertion.

There are some limitations of the present study. Firstly, the majority (77.6%) of patients underwent diagnostic colonoscopy, which may limit its extrapolations. However, subgroup analysis showed that DCS was also effective in patients undergoing screening or surveillance colonoscopy. Secondly, this study was performed in patients with unsedated status. So there may be some confounding factors, such as levels of anxiety, the tolerance of pain or discomfort, directly associated with insertion time. Thirdly, our study only enrolled one group of patients as the training cohort for establishing the model. The external validation in another independent validation cohort was absent. Fourthly, air was insufflated instead of CO2 during colonoscopy. It will be interesting to investigate whether CO_2_ has potential impact on DCS in another study. Finally, the generalizability of this study may be limited by the clinical setting in which the examination were performed in only one tertiary center. Therefore, to avoid these influences, the conclusion needs further validation.

## Conclusions

In summary, advanced age, lower BMI, inexperienced operator and relatively poor sleep condition were associated with longer insertion time, and we developed a novel, objective, noninvasive and conveniently applicable predictive score (DCS) to prejudge the potentially difficulty colonoscopy in preoperational stage.

## Additional files

Additional file 1: Figure S1. Kaplan-Meier curves of insertion time in patients with different BMI. Additional file 2: Figure S2. ROC analysis for the prediction of patients with insertion time less than 10 min by DCS.
