# Supplementary figures and images for: Difficult colonoscopy score identifies the difficult patients undergoing unsedated colonoscopy
Source: BMC Gastroenterol. 2015 Apr 9;15:46. doi: 10.1186/s12876-015-0273-7 (PMC4397830; doi:10.1186/s12876-015-0273-7)

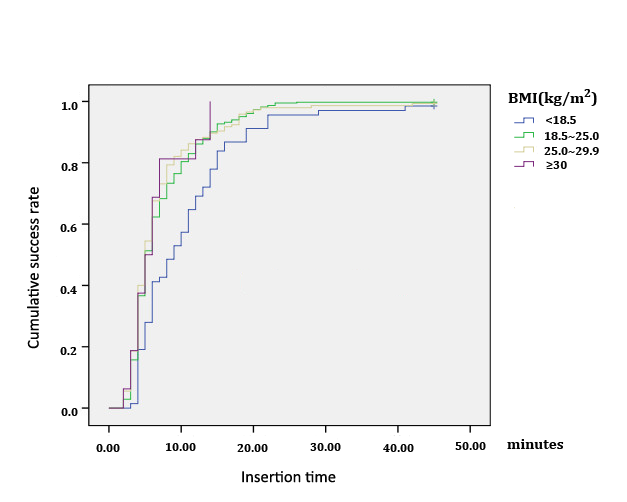

Supplement: Additional file 1: Figure S1. — Kaplan-Meier curves of insertion time in patients with different BMI. [file 12876_2015_273_MOESM1_ESM.tiff]

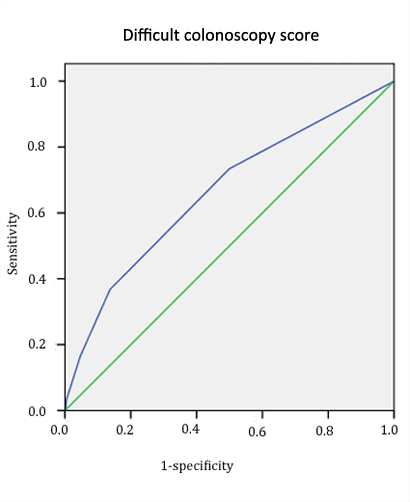

Supplement: Additional file 2: Figure S2. — ROC analysis for the prediction of patients with insertion time less than 10 min by DCS. [file 12876_2015_273_MOESM2_ESM.tiff]
